# Evaluation of the Epidemiological Disease Burden and Nationwide Cost of Endometriosis in Hungary

**DOI:** 10.3390/healthcare12242567

**Published:** 2024-12-20

**Authors:** Dalma Pónusz-Kovács, Róbert Pónusz, Luca Fanni Sántics-Kajos, Tímea Csákvári, Bettina Kovács, Ákos Várnagy, Kálmán András Kovács, József Bódis, Imre Boncz

**Affiliations:** 1 Institute for Health Insurance, Faculty of Health Sciences, University of Pécs, Vörösmarty Street 3, 7621 Pécs, Hungary; ponusz.robert@pte.hu (R.P.); luca.santics-kajos@etk.pte.hu (L.F.S.-K.); timea.csakvari@etk.pte.hu (T.C.); kovacs.bettina@pte.hu (B.K.); boncz.imre@pte.hu (I.B.); 2 National Laboratory on Human Reproduction, University of Pécs, Vasvári Pál Street 4, 7622 Pécs, Hungary; varnagy.akos@pte.hu (Á.V.); kovacs.kalman@pte.hu (K.A.K.); bodis.jozsef@pte.hu (J.B.); 3 Department of Obstetrics and Gynecology, Medical School, Clinical Center, University of Pécs, Édesanyák Street 17, 7624 Pécs, Hungary

**Keywords:** endometriosis, age-standardized prevalence, cost of illness, economic burden

## Abstract

Background: Endometriosis is one of the most common gynecological diseases that can lead to infertility. The aim of this quantitative, descriptive, and cross-sectional study was to analyze the prevalence and the annual nationwide health insurance treatment cost of endometriosis in Hungary in 2010 and 2019. Methods: The data used in this study were sourced from publicly funded, national, real-world datasets administered by the National Health Insurance Administration (NHIFA). The total number of cases of endometriosis in the Hungarian population was determined by ICD codes and all types of care. The total prevalence, age-specific prevalence, and annual health insurance expenditure by age group were evaluated. Results: The highest numbers of patients and prevalence (2010: 101.9/100,000 women; 2019: 197.3/100,000 women) were found in outpatient care. Endometriosis, regardless of its type, mainly affects patients in the 30–39-year age group (number of patients—2010: 6852; 2019: 11,821). The NHIFA spent a total of EUR 1,639,612 on endometriosis treatment in 2010 and EUR 1,905,476 in 2019. The average annual health insurance expenditure per capita was EUR 574 in 2010 and EUR 426 in 2019. There was a significant correlation between length of stay and mean age of patients in both years (2010 r = 0.856, *p* < 0.001; 2019 r = 0.877, *p* < 0.001). Conclusions: The number endometriosis cases is increasing. Early diagnosis and targeted treatment would reduce endometriosis symptoms and therefore improve patients’ quality of life and reduce health insurance costs. This would be helped by the establishment of endometriosis centers.

## 1. Introduction

Endometriosis is a chronic gynecological condition associated with the presence of endometriosis-like tissue outside the uterus [1]. Its etiology is complex, most often due to immune dysfunction, metaplasia, hematogenous or endogenous endometrium proliferation, and persistent low-grade inflammation [2,3,4,5]. Lifestyle factors can also contribute to the development of endometriosis, including a sedentary lifestyle, chronic stress, poor dietary habits, and previous abdominal trauma [6,7]. Endometriosis could be associated with a family history of genetic predisposition in 7% of women [6]. Apart from being associated with pain and other unpleasant symptoms for patients, endometriosis is also one of the most common causes of infertility [8,9,10]. The presence of the disease can affect many aspects of life: it can have a significant impact on quality of life (QoL), affect learning performance, and social relationships, and it can also cause depressive symptoms, anxiety, and psychosocial stress [11,12,13]. Depending on the severity of the symptoms, not only can the direct medical cost be a significant burden, but the indirect cost of the disease can also have a serious impact on the affected persons’ life due to the loss of working productivity and sick leave [14]. The disease affects about 18% of women of reproductive age globally, but rates vary by continent: 36% in Asia, 26% in Africa, 19% in the United States, and 19% in Europe [15]. The American Society of Reproductive Medicine (ASRM) classification system distinguishes four stages of endometriosis: stages I (minimal) and II (mild) have deep lesions and thin adhesions that are absent or rare, while stages III (moderate) and IV (severe) can develop larger adhesions and endometriomas and possibly spread to other organs [16], further worsening the patient’s quality of life (QoL) [17].

The diagnosis and treatment of endometriosis require the involvement of many areas of the healthcare system [18]. In addition, patient awareness and socio-cultural factors related to pain management are very important [19]. Specific diagnosis usually takes years due to nonspecific symptoms [15,20]; moreover, the average diagnosis time varies from country to country [21]. In Europe, the average delay from the onset of symptoms to diagnosis is 10.4 years [22], 11.0 years in Italy [15], 3.9 years in Hungary [23], and 3.5 years in the Netherlands [24]. The average time is significantly affected by whether the patient is treated in a public or private healthcare system [25].

The treatment of the disease is complex, but the dominant approach is symptomatic therapy: pain relief is an important therapeutic goal in the initial phase, followed by an invasive procedure called laparotomy to remove the enlarged mucosal tissue. The use of hormone replacement therapy and the treatment of psychological symptoms complements this [26,27]. The prevalence of endometriosis is increasing in developed societies [28]; the social costs of illness vary from country to country but are on an upward trend [29,30]. The demographic challenges in Europe and the traditionally strong role of the public sector in the organization and financing of healthcare on the continent are raising awareness of the importance of prevention, timely diagnosis, adequate treatment, and improved quality of life. Through these areas, the burden of disease on individuals and the costs to society can be reduced [31].

### The Healthcare System in Hungary

The Hungarian healthcare system is also highly centralized and state-dominated in terms of resource generation, financing, and service provision [32]. The health insurance system follows the Bismarckian tradition (i.e., a social security contribution is the basis for access to services, to which citizens are obliged to subscribe) [33]. The working-age population is liable to pay social security contributions, while contributions for students and pensioners or other vulnerable groups are covered by the central budget. The bulk of the financing of health services is provided by the compulsory health insurance scheme, which covers 95% of the population [34]. Social security contributions cover 2/3 of public health expenditure; the remaining source is typically made up from government funds (taxes) and direct contributions (co-payments) from the population [32].

Any healthcare provider, regardless of ownership, can initiate a financing contract with the National Health Insurance Fund Administration (NHIFA), but mainly public (governmental) and non-profit (church) groups have contracts. Providers report monthly on the volume and services covered by the contract in the form of performance reports. The most important source of revenue for providers is linked to the activities of the public health system, which comes from the NHIFA. Private household (out-of-pocket payment; OOP) expenditure is an increasingly important source of health expenditure, accounting for 26% of total health expenditure in 2010 and 27% in 2019 [35,36]. The role of private health insurers is complementary and mainly covers the provision of amenities (rooms, catering, etc.). Only a small proportion of the Hungarian population has private health insurance. The regulation, functioning, and structural features of the Hungarian healthcare system have been described in detail elsewhere [12,37].

Most of the endometriosis-related scientific articles focus on certain treatments, diagnostic approaches, or cost-effectiveness, using data mainly from small-scale clinical studies; thus, there is a lack of studies within the international academic literature that are based on a nationwide, real-world clinical dataset [38,39].

The aim of the study was to determine the prevalence of endometriosis based on the data of the entire Hungarian publicly funded healthcare system independently from the type of the caregiver institution and, furthermore, to compare the annual health insurance expenditure associated with endometriosis in 2010 and 2019.

## 2. Materials and Methods

We performed a retrospective database analysis using the nationwide, real-world database of the Hungarian National Health Insurance Fund Administration containing the healthcare data of all publicly financed healthcare providers in Hungary. Utilizing this nationwide database, covering the whole Hungarian population and all the publicly financed healthcare providers, allowed us to perform unique analyses.

The research analyzed a nationwide database of the real-world data of all publicly funded healthcare providers in Hungary for the years 2010 and 2019, provided by the NHIFA, the only public healthcare financing agency in Hungary [40]. The patients with endometriosis included in the study were classified according to the World Health Organization International Classification of Diseases (WHO ICD, 10th revision) and identified as “endometriosis of the uterus” (N80.0), “endometriosis of the ovary” (N80.1), “endometriosis of the fallopian tubes” (N80.2), “endometriosis of the pelvic peritoneum” (N80.3), “endometriosis of the rectovaginal septum and vagina” (N80.4), “other endometriosis” (N80.8), and “endometriosis unspecified” (N80.9).

The analysis covered all healthcare providers and forms of care financed by the NHIFA, including general practitioner care (GP), home care, nursing, patient transport, ambulance, laboratory diagnostics, diagnostic imaging (CT, MRI, and PET), acute and chronic inpatient care, subsidized medicaments, and subsidized medical aids.

To determine the number of patients with endometriosis and the health insurance costs of treatment, we included diagnoses that appear as the main diagnosis justifying care in active and chronic inpatient care. For discharge, only data on patient flows were available. No patient turnover was recorded within PET diagnosis.

To determine the epidemiological characteristics of the diseases included in the study, we examined the annual number of patients and the prevalence per 100,000 in the female population, both by age group and by type of treatment. For the age groups, 7 groups were defined: 0–19 years, 20–29 years, 30–39 years, 40–49 years, 50–59 years, 60–69 years, and over 70 years. The prevalence was analyzed for the type of care with the highest number of patients, which was outpatient care for all the above diagnoses. Prevalence was calculated with the standardized value for age. Data for the Hungarian population were derived from the 2010 and 2019 Hungarian Central Statistical Office (HCSO) databases [41]. Age-standardized prevalence rates were calculated using the European Standard Population 2013 (ESP2013).

Concerning the health insurance burden of disease, we determined the annual expenditure and distribution of each pathology as well as the amount of NHIFA expenditure per patient. These costs are detailed by age group and by type of care.

The costs are expressed in euro (EUR), using the mean exchange rate of the Hungarian National Bank in 2010 and 2019 (2010: EUR 1 = HUF 188.56; 2019: EUR 1 = HUF 325.35) [42].

The data were processed using SPSS 27.0.1 (Statistics Pack-Age for Social Sciences 27.0.1) and Microsoft Office 2016 Excel, and statistical analysis was performed using paired linear regression and *t*-test. The goal of the chosen statistical tests was to determine the significant relationship between the variables (average age and average per capita health expenditure) and to set the degree of correlation. Values are presented at the 5% (*p* < 0.05) significance level.

## 3. Results

The number of cases diagnosed with endometriosis in the publicly funded healthcare system, the average age of patients, and the health insurance expenditure also showed an increasing trend in the analyzed period. The indicators in accordance with healthcare services are presented in Table 1.

A review of the various forms of care revealed that the highest patient turnover was observed in outpatient care [2010 n = 12,543 (41.6%); 2019 n = 22,516 (38.0%)]. This was followed by GP [2010: n = 8029 (26.6%); 2019: n = 18,935 (31.9%)] and subsidized medicaments treatments (2010: n = 6352 (21.0%); 2019: n = 11,936 (20.1%)]. In 2010, these three forms of care covered 89.2% of the total sample’s patient turnover; in 2019, this proportion was 90.1%.

A three-times higher annual number of cases was registered in 2019 related to CT and MRI diagnostics compared to 2010 (+773 cases). A similar tendency was captured both in outpatient care (1.8-times higher number of cases) and subsidized medicaments (1.9-times higher number of cases, +5581 cases).

The mean age of patients in 2010 was 38.3 years (95% CI: 34.3–42.2), while in 2019, it almost reached 40 years (39.6; 95% CI: 36.3–42.9). The highest mean age was registered in 2010 for patient transport (75.3 years), followed by patients supplied with medical aids (53.7 years), patients in CT or MR diagnostics (38.9 years), and patients with subsidized medicaments (37.8 years). In 2019, the mean age of patients also increased in every aspect except the CT and MR diagnostics and medical aids (55.6 years). The most marked increases in mean age were observed in the areas of home care (+5.8 years), chronic inpatient care (+5.5 years), and outpatient care (+2.7 years).

In 2010, NHIFA funded the treatment of patients diagnosed with endometriosis with a total of EUR 1,639,613. In 2019, the amount of funding was 16.2% higher, reaching almost EUR 2 million (EUR 1,905,476).

Both in 2010 and in 2019, the cost of subsidized medications comprised the largest share of healthcare expenditure. In 2019, 14.8% higher expenditure was observed in the costs related to subsidized medications (EUR +119,005). Besides medications, the costs of inpatient and outpatient care were also significant. The share of endometriosis-related expenditures of subsidized medications as well as inpatient and outpatient care represented 90.8% of the total expenditure for treatment in 2010 and 86.0% in 2019.

Table 2 illustrates that the highest patient turnover was related to “endometriosis, unspecified” (ICD N80.8). In 2010, it covered 60.2% of the total number of cases, and in 2019, it grew to 69.8%. In defining patient pathologies, “endometriosis of the uterus” (ICD N80.0; 2010: 13.3%; 2019: 16.8%) and “ovarian endometriosis” (ICD N80.1; 2010: 16.8%; 2019: 13.9%) were represented in almost the same proportion, while other types of endometrioses represented a significantly lower share.

The number of inpatient cases in 2019 was 12% higher than in 2010 (+129 cases). The mean length of stay (LoS) for patients with endometriosis was 3.9 days in 2010, which decreased to 2.8 days in 2019.

From a diagnosis-specific point of view, patients with “endometriosis of the uterus” (ICD N80.0) had the longest length of stay (5.5 days), followed by “endometriosis of the ovary” (ICD N80; 14.2 days) in 2010. In 2019, “endometriosis of the uterus” (ICD N80.0) also had the longest LoS (5.3 days), followed by “endometriosis of the pelvic peritoneum” (ICD N80.3; 2.9 days). In both years of the study, a significant correlation was observed between LoS and the mean age of patients (2010: r = 0.856, *p* < 0.001; 2019: r = 0.877, *p* < 0.001).

The NHIFA funded the treatment of “endometriosis, unspecified” (ICD N80.9) with EUR 859,489, which covered 52.4% of the total endometriosis expenditure in 2010. In 2019, the level of public funding increased up to EUR 1,277,945, which contributed to an even more significant ratio of diagnosis-specific expenditure (67.1%).

The second highest level of health insurance expenditure was related to “endometriosis of ovary” (ICD N80. 1). It represented EUR 429,541 in 2010 and decreased to EUR 409,157 in 2019. Treatment costs for other endometriosis diagnoses represented a more moderate expenditure for NHIFA.

On average, the highest per-patient health insurance expenditure, calculated based on the subsidized medicaments, was for “endometriosis of ovary” (ICD N80.1) in both years under review: in 2010, it represented EUR 1220/patient; in 2019, it decreased to EUR 967/patient.

In the diagnosis categories “endometriosis of the rectovaginal septum and vagina” (ICD N80.4), “other endometriosis” (ICD N80.8), and “endometriosis, unspecified” (ICD N80.9), the main health insurance expenditure was related to subsidized medicaments funding during the study period. It is important to highlight that for “endometriosis of uterus” (ICD N80.0), “endometriosis of ovary” (ICD N80.1), “endometriosis of fallopian tube” (ICD N80.2), and “endometriosis of pelvic peritoneum” (ICD N80.3), the most significant expenditure was dedicated to inpatient care.

**Figure 1:**
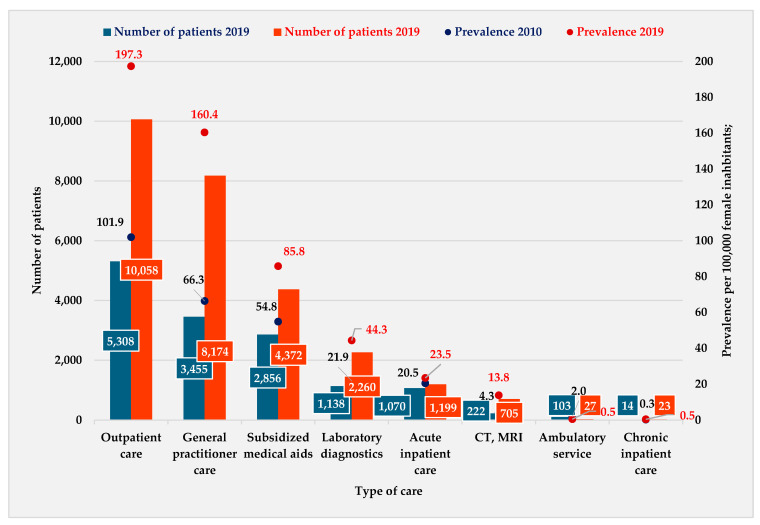
Distribution of the number of patients and prevalence per 100,000 female inhabitants by type of care (NHIFA, 2010, 2019).

Figure 1 shows the prevalence of endometriosis per 100,000 female inhabitants was 273.1 in 2010 and 527.4 in 2019. The type of treatment had a significant impact on the prevalence during the whole study period. In both 2010 and 2019, the highest prevalence was registered in outpatient care, followed by GP care and subsidized medicaments. A similar prevalence was observed in GP patient turnover in 2019 compared to 2010 (+94.1 patients/100,000 female population) and in outpatient care (+94.1 patients/100,000 female population) (Figure 1).

The number of visits per patient was highest in the following types of care: GP (2.3 visits per patient in 2010; 2.3 visits per patient in 2019), outpatient care (2.4 visits per patient in 2010; 2.2 visits per patient in 2019), as well as subsidized medicaments (2.2 visits per patient in 2010; 2.7 visits per patient in 2019).

**Figure 2:**
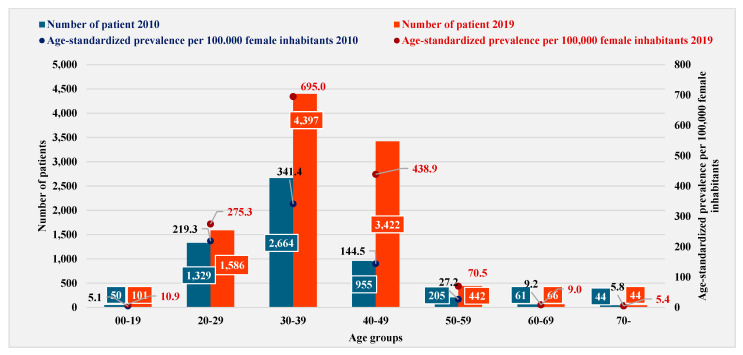
Distribution of number of patients and age-standardized prevalence per 100,000 female inhabitants by age group (NHIFA 2010, 2019).

According to the historical data of endometriosis patient turnover, which is captured in Figure 2, the most affected age group was 30–39 years in both studied years (2010: 50.2%; 2019: 43.7%). In 2010, the 20–29-year age group represented 25.0% of the total turnover, while the 40–49-year age group accounted for 18.0%. In 2019, however, this trend reversed: the 40–49-year age group represented the second highest age group of the total turnover (34.4%).

The prevalence rates were standardized using data derived from the European Standard Population 2013 (ESP2013) [43]. The prevalence standardized by age was 101.9 (1.0%) in 2010, while in 2019, it was 197.3 (2.0%) per 100,000 female population. In 2019, a similar increasing trend was observed for the standardized prevalence, with the highest value calculated for the 30–39-year age group (2.0 times higher than in 2010), followed by the 40–49-year age group (3.0 times higher in 2010) and then the 20–29-year age group with a more moderate increase (1.2 times higher in 2010).

**Figure 3:**
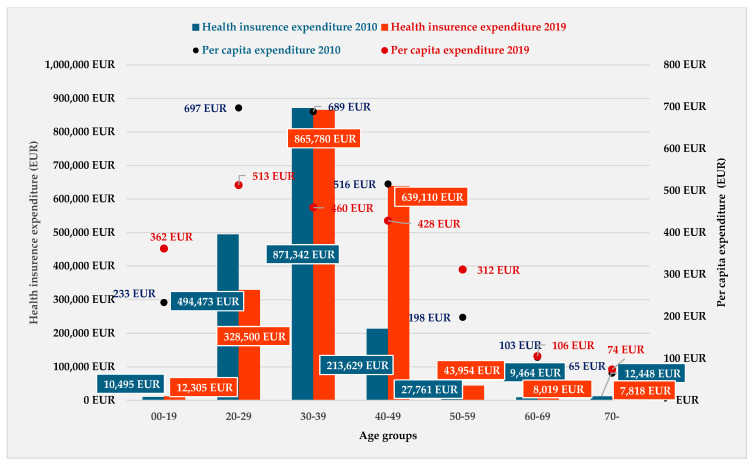
Distribution of total health insurance expenditure and per capita expenditure by age group (NHIFA, 2010, 2019).

Figure 3 illustrates the cost of treatment of endometriosis. In 2010, the treatment costs were the highest in the age group of 30–39 years, which was followed by the costs of the age group of 20–29 years and 40–49-year age group. In 2019, treatment costs were the highest in the age group of 30–39 years, followed by the age group of 40–49 years and then by the 20–29-year age group.

The total amount of health insurance expenditure by the type of treatment was the highest in subsidized medicaments. The health insurance expenditure per patient was EUR 574 in 2010, while in 2019, it was lower, at EUR 426 per patient.

By age group, the highest per capita expenditure in 2010 was calculated for the 20–29-year age group (EUR 697), followed by patients aged 30–39 (EUR 689). In 2019, the health insurance expenditure represented a slightly lower cost compared to 2010 (−25.8%). The highest costs were among the 20–29-year age group, followed by 30–39- and 40–49-year-old patients.

On the one hand, the subsidized medicaments represented the highest health insurance expenditure in the study period; on the other hand, the highest cost per patient was for inpatient care. In 2010, the cost per patient was EUR 1526, while in 2019, it was approximately the same (EUR 1554) (Figure 3).

A correlation analysis for 2010 showed a significant relationship between average age and per capita healthcare expenditure on laboratory diagnostics (r = 0.853, *p* < 0.001). This result demonstrates that the expenditure on laboratory services increases with the age of the patient. In 2019, significant associations for nursing home care (r = 0.945, *p* < 0.001) and chronic inpatient care (r = 0.973, *p* < 0.001) in the per capita expenditure and the age of the patients were captured. Despite a significant association in both years examined, a negative correlation was found between health insurance expenditure on subsidized medications and age of patients with endometriosis. The results imply that younger patients’ medication costs were higher for the national health insurers company (Table 3).

## 4. Discussion

By analyzing the routinely collected real-world data from the NHIFA database for 2010 and 2019, we determined the epidemiological and financing characteristics of endometriosis in Hungary, broken down by different types of care and age groups. The study did not require an ethics clearance request, as the database did not contain any personal health data and did not allow for the identification of patients.

Based on the annual data of publicly funded care in Hungary, we found that the prevalence of endometriosis was 2.73% in 2010, and it slightly increased to 5.1% in 2019. Similar to the international examples, it is important to highlight that the increasing tendency in prevalence of endometriosis could be deduced from the development of diagnostic techniques. In Western countries, it is a common phenomenon that the patients have a developed health-conscious attitude, so they attend more physician visits. The prevalence of endometriosis varies widely: its value is influenced by the size of the population, the patient age, or the development of the country. According to some international studies, endometriosis ranges from 0.7 to 8.6% of the total population [44,45,46], which was confirmed by the results of our research. It is important to note that other papers have reported higher prevalence values [9,38,47]. One of the limitations of our study is that we were only able to analyze the annual burden of disease for endometriosis in the publicly funded healthcare system. We did not have information on the number of patients diagnosed and treated in the private sector and the amount of their private reimbursement. It can be assumed that the number of patients and cases registered and the healthcare expenditure associated with endometriosis in Hungary may represent a higher ratio in everyday practice.

The exact prevalence of the disease cannot be determined; it is calculated from laparoscopic findings, and its actual prevalence is assumed to be higher than the ratios reported in the literature [48]. One of the most prominent reasons behind the discrepancy is diagnostic delay [10,15,41,49]. The exact prevalence in our study may also represent a higher value in everyday practice, as the utilization of private and for-profit gynecological practices is one of the highest among specialist services, but the privately held and funded caregivers are not obliged to report patient turnover to the NHIFA [50].

The results of our study also confirm that women of reproductive age have the highest prevalence of endometriosis: in both 2010 and 2019, the age group of 30–39 years was the most affected (2010: 101.9 patients/100,000 female population; 2019: 197.3 patients/100,000 female population). This age group showed a 3.3-fold increase in prevalence, similar to the results reported by other studies [51,52]. As women increase in age, there could be biological barriers to conception, which often require medical attention. In many cases, the presence of endometriosis is verified in that phase.

There are also results within the international literature that show the highest prevalence of endometriosis is in the 40–49-year age group [53]. Our results from the nationwide real-world database cannot confirm this, but for 2019, the 40–49-year age group showed a significant increase (+295 patients/100,000 female population), making it the second most affected age group compared to 2010 [9,54,55]. The average age of the patients also showed an upward trend, indicating that women of reproductive age are not the only ones affected [56].

According to the literature, MR imaging is considered the most reliable test for diagnosing endometriosis and determining its precise location [57,58,59,60,61]. This also correlates with our own results, as the prevalence calculated on the basis of CT and MRI care patient-flow data showed the most significant, 3.2-times increase in 2019. The number of cases in 2019 almost doubled compared to 2010 in terms of the number of patients and number of cases. The most significant increases were in general practitioner (+135.8%, +10,906 cases) and outpatient care (+79.5%, +9973 cases). These treatments are the less cost-demanding treatments; thus, with the nature of the cost structure of the aforementioned treatments, this would be the most solid factor explaining why total expenditure did not change at a similar pace as the number of cases in 2019.

Compared to certain international results, where 68% of patients visited their GP one or more times a year, there has been a lag in the number of visits to GPs in Hungary despite an increase in number of patients [62]. In the years studied, nearly one-third of the cases visited a GP (24.3% in 2010; 30.4% in 2019), while in both years, there was an average of 2.32 visits per patient per year. We report a negative correlation between average age and the number of GP visits per year, with younger patients visiting their GP more often compared to older patients, similar to findings in Sweden [49]. Similar results have been reported for outpatient care, laboratory, and medical imaging diagnostics. Inpatient care typically showed rates indicative of under-utilization in our study sample [63,64]. In line with international practice, by 2019, the length of hospital stay for treated patients fell to an average of 2.8 days compared to more than 3 days 10 years earlier [23,65,66,67].

The annual health insurance expenditure on endometriosis in 2019 was EUR 1.64 million, which is 14.4% higher than it was in 2010. A similar analysis in the U.S. reported a much higher total expenditure and a 42.2% increase over the last decade [27]. Although the prevalence of endometriosis has increased, there has been no evidence of a more developed increase in health insurance costs due to the fact that the health insurance financing system in Hungary has not kept pace with the rising costs in clinical practice. The highest utilization and health insurance expenditure was associated with the diagnosis of WHO ICD N80.9 (“endometriosis, unspecified”). Compared to the 2010 baseline, health insurance expenditure for the treatment of this diagnosis increased by 48.7%. The high prevalence of WHO ICD N80.9 is supported by some studies and clinical practice, as the lack of detection of the exact site of endometriosis and coding inaccuracies can have a biasing effect when establishing the diagnosis [57]. According to the pathologies of endometriosis, WHO ICD N80.1 (“endometriosis of the ovary”) had the highest treatment costs in the years studied. A Canadian study with a similar study purpose highlighted the same result [49]. The highest per capita costs in both years were in inpatient care (2010: EUR 526/patient; 2019: EUR 1554/patient) [68]. The number of hospital days decreased, while the number of cases increased modestly in 2019; however, healthcare expenditure stagnated in Hungary, while in other countries, it increased year after year [69]. The annual inpatient expenditure per patient was similar to the costs in Canada, but the direct costs in the U.S. were five times higher [70]. It is well known that health insurance expenditure is typically adversely affected by diagnostic delays [71]. In addition, it is important to highlight that the cost of the treatment of pain, anxiety, or infertility associated with endometriosis could be increasing, which can put more pressure on the individuals and the financing bodies as well [72]. In Steven’s study, 10% of the costs of the treatment were related to pharma-related expenditures, but this was much lower than in our database, where it was nearly 50%. Despite a reduction in per-patient pharma costs, by 2019, the annual per-patient expenditure rate was higher than reported in the literature [63]. Not just in Hungary but also in the international findings, the direct costs per capita were higher for the 20–29-year age group than for patients above 30 years old [24].

Despite the increase in the overall number of cases, the most pronounced increase was in the age groups requiring lower treatment costs, while for age groups with higher direct treatment costs, as in other countries, a decrease in the number of cases was found (age groups 0–19; 20–29) [24,58]. As opposed to international practice, there was no increase in the expenditure on surgical procedures, and a decrease in the cost of medication per patient in 2019 was registered; however, the annual expenditure per patient was higher in Hungary than that reported in the academic literature [28].

A further limitation is that the direct healthcare expenditure associated with the diagnosis of endometriosis is described in our study, which shows an increasing tendency every year. Given its multifactorial origin and holistic medical practice, the analysis of indirect expenditure related to the disease, for which our study database did not contain data, cannot be neglected.

## 5. Conclusions

Our research also supports the fact that the age of patients diagnosed with endometriosis is on the rise. As one of the most common gynecological diagnoses, it can occur from adolescence through reproductive age and even beyond menopause. There is an increasing need to develop systems that can support caregivers in the early detection of endometriosis as well as enhance the consciousness of the patients. Nowadays, education for adolescents about endometriosis, pregnancy and reproductive health, or about post-menopausal endometriosis is becoming increasingly important. Strengthening the role and the capacities of general practitioners as the first line of medical attention could be crucial as well. In line with international practice, the establishment of endometriosis centers in Hungary would have paramount importance in the future to enhance the support of clinical care and research activities. These centers would significantly improve the speed of diagnosis and timely, targeted, complex therapy as well as enabling the decision makers to allocate the levels of funding and clinical resources that endometriosis treatment requires. This research could help in the identification of the causes of endometriosis, the elucidation of its incidence and prevalence, and in highlighting the need for the development of new drugs and non-invasive biomarkers for providing adequate and effective therapies.

Our nationwide study on endometriosis fulfills a significant need from both an epidemiological and an economic point of view. The study highlights the importance of the problem of this growing and increasingly costly gynecological phenomenon. Our research may also help in the preparation of future health policy and resource allocation decisions.

## Figures and Tables

**Table 1:** Distribution of average age, number of cases, and health insurance expenditure by type of care [(National Health Insurance Fund Administration NHIFA), 2010, 2019].

Type of Care	Mean Age		Number of Cases		Health Insurence Expenditure (EUR)		Distribution of Health Insurance Expenditures (%)	
	2010	2019	2010	2019	2010	2019	2010	2019
General practitioner care	37.6	38.4	8029	18,935	85,481	116,835	5.2%	6.1%
Home care	--	36.4	--	3	--	433	--	0.0%
Patient transportation	75.3	72.8	89	93	1040	1748	0.1%	0.1%
Outpatient care	34.9	37.5	12,543	22,516	87,186	119,009	5.3%	6.2%
Care in care centres	35.2	41.0	152	51	1151	489	0.1%	0.0%
Laboratory diagnostics	35.3	36.6	1513	3305	8716	16,249	0.5%	0.9%
CT, MRI	38.9	38.2	394	1167	46,087	110,441	2.8%	5.8%
Acute inpatient care	34.5	35.8	1080	1220	603,116	602,787	36.8%	31.6%
Chronic inpatient care	31.2	36.7	14	23	6216	18,338	0.4%	1.0%
Subsidized medicaments	37.8	38.8	6352	11,936	799,412	918,417	48.8%	48.2%
Subsidized medical aids	53.7	55.6	11	16	1208	730	0.1%	0.0%
Total	--	--	--	--	1,639,613	1,905,476	100.0%	100.0%

**Table 2:** Number of cases and health insurance expenditure for different diagnosis of endometriosis (NHIFA, 2010, 2019).

	N80.0		N80.1		N80.2		N80.3		N80.4		N80.8		N80.9			
Type of Care	Uterus		Ovaries		Fallopian Tube		Pelvic Peritoneum		Rectovaginal Septum, Vagina		Other		Unspecified		Total	
	2010	2019	2010	2019	2010	2019	2010	2019	2010	2019	2010	2019	2010	2019	2010	2019
General practitioner care	1305	2288	1834	3369	100	192	418	618	70	127	427	796	3875	11,545	8029	18,935
Home care	--	0	0	0	0	0	0	0	0	0	0	0	0	3	0	3
Patient transportation	50	56	12	6	5	0	4	0	0	0	9	10	9	21	89	93
Ambulatory service	1	10	2	1	0	0	0	0	0	1	0	2	9	9	12	23
Outpatient care	1109	1478	1801	3009	64	90	250	425	171	130	622	614	8526	16,770	12,543	22,516
Care in care centres	33	13	16	7	0	0	0	0	0	1	6	0	97	30	152	51
Laboratory diagnostics	235	263	190	387	6	18	24	37	16	16	80	98	962	2.486	1513	3305
CT, MRI	54	111	63	121	1	5	16	35	7	3	26	46	227	846	394	1.167
PET	0	0	0	0	0	0	0	0	0	0	0	0	0	0	0	0
Acute inpatient care	64	90	504	477	1	10	78	63	23	5	101	57	309	518	1080	1220
Chronic inpatient care	9	0	2	4	0	0	2	3	0	1	0	1	1	14	14	23
Disposable instruments, implantations, and medicaments falling under itemized accounts	0	0	0	0	0	0	0	0	0	0	0	0	0	0	0	0
Subsidized medicaments	1144	1493	633	831	47	17	89	167	34	55	259	232	4146	9141	6352	11,936
Subsidized medical aids	7	2	0	1	0	0	0	0	3	4	0	3	1	6	11	16
Maximum value	1305	2288	1834	3369	100	192	418	618	171	130	622	796	8526	16,770	12,543	22,516

**Table 3:** Own calculations based on data from the NHIFA (NHIFA, 2010, 2019).

Type of Care	Mean Age		Health Insurance Expenditure per Patient (EUR)		Mean Age vs. Health Insurance Expenditure per Patient 2010		Mean Age vs. Health Insurance Expenditure per Patient 2019	
	2010	2019	2010	2019	r-Value	*p*-Value	r-Value	*p*-Value
General practitioner care	37.6	38.4	24.7	14.3	0.245	0.250	−0.007	0.973
Home care	--	36.4	--	216.5	--	--	--	--
Patient transportation	75.3	72.8	29.7	62.4	--	--	0.511	0.018
Ambulatory service	43.9	40.5	0.0	--	--	--	--	--
Outpatient care	34.9	37.5	16.4	11.8	−0.173	0.413	−0.030	0.885
Care in care centres	35.2	41.0	11.2	18.1	--	--	0.945	0.000
Laboratory diagnostics	35.3	36.6	7.7	7.2	0.853	0.000	−0.503	0.020
CT, MRI	38.9	38.2	207.6	156.7	−0.307	0.151	0.178	0.401
PET	--	--	--	--	-	--	--	--
Acute inpatient care	34.5	35.8	563.7	502.7	0.323	0.131	0.405	0.060
Chronic inpatient care	31.2	36.7	444.0	797.3	--	-	0.973	0.000
Disposable instruments, implantations, and medicaments falling under itemized accounts	--	--	--	--	--	--	--	--
Subsidized medicaments	37.8	38.8	279.9	210.1	−0.272	0.202	−0.356	0.097
Subsidized medical aids	53.7	55.6	151.0	66.4	--	--	0.011	0.957

## Data Availability

Data were obtained from the National Health Insurance Fund Administration (NHIFA) of Hungary and are available from the authors with the permission of the NHIFA. Restrictions apply to the availability of these data.
